# Neurovascular unit remodeling: focusing on glial cells in stroke injury and recovery

**DOI:** 10.3389/fncel.2026.1821227

**Published:** 2026-05-14

**Authors:** Meng Gong, Xinyi Li, Qiang Yuan, Lina Pang, Xiangyin Ye, Shufang Li, Yan Li, Hong Guo, Song Jin

**Affiliations:** 1 School of Acupuncture and Tuina, Chengdu University of Traditional Chinese Medicine, Chengdu, China; 2 School of Health Preservation and Rehabilitation, Chengdu University of Traditional Chinese Medicine, Chengdu, China; 3 Department of Tuina, Hospital of Chengdu University of Traditional Chinese Medicine, Chengdu, China; 4 School of Medical and Life Sciences, Chengdu University of Traditional Chinese Medicine, Chengdu, China; 5 Department of Rehabilitation, Hospital of Chengdu University of Traditional Chinese Medicine, Chengdu, China

**Keywords:** glial cell, neurovascular unit, pathophysiology, stroke, therapeutics

## Abstract

Stroke stands as a predominant global contributor to mortality and persistent disability, marked by a sequential cascade of pathophysiological perturbations. Moving beyond the traditional neuron-focused paradigm, contemporary stroke research has increasingly centered on the neurovascular unit (NVU), a functional multicellular assembly encompassing neurons, glial cells, and cerebrovascular components, as the pivotal regulatory hub for cerebral homeostasis and adaptive responses to injury. Among NVU components, glial cells are no longer viewed as passive supporters but as active regulators of NVU integrity, blood–brain barrier (BBB) functionality, immune surveillance, and tissue repair processes. Stroke trigger distinct spatiotemporal phenotypic changes in glial cells. Microglia show biphasic pro-inflammatory and pro-repair polarization. Astrocytes undergo reactive gliosis and form glial scars with dual functions. Oligodendrocytes experience iron-related or ischemic injury and contribute to remyelination. Hypertension, hyperglycemia and aging jointly induce glial dysfunction and predispose the NVU to a pre-vulnerable state that worsens post-stroke damage. This review emphasizes the multifaceted roles of glia-mediated NVU remodeling in stroke injury and recovery, addresses the modulatory effects of comorbid risk factors on glial functions and explores glia-targeted therapeutic strategies that hold promise for preserving NVU function and improving stroke outcomes.

## Graphical abstract

**Figure:**
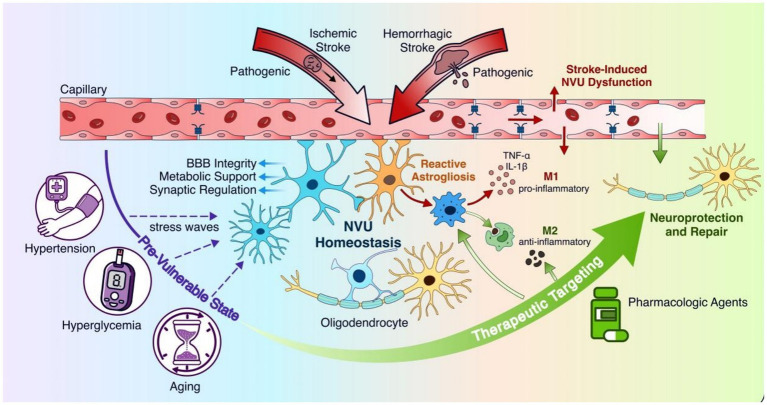

## 1 Introduction

Stroke remains a leading cause of death and long-term disability worldwide. Its pathogenesis involves a rapid cascade of events, including calcium overload, glutamate excitotoxicity, oxidative stress, neuroinflammation, mitochondrial dysfunction, and apoptosis, ultimately leading to irreversible neuronal loss (Chen C. et al., 2019). Previous reviews have summarized that the previous research has predominantly focused on neurons as the primary targets of stroke. However, this neuron-centric perspective fails to encompass the integrated cellular network that governs cerebral homeostasis and the response to injury (Garcia-Martínez et al., 2025). Recognizing this limitation, the Stroke Progress Review Group of the National Institute of Neurological Disorders and Stroke formally introduced the concept of the neurovascular unit (NVU) in 2001 (Iadecola, 2017). The NVU is a functional multicellular complex composed of neurons, glial cells, and cerebrovascular elements. Among these, glial cells are now recognized not merely as passive supporters but as active regulators of NVU integrity, blood–brain barrier (BBB) dynamics, immune surveillance, and tissue repair (Zhang X. et al., 2019).

In stroke, glial cells undergo profound phenotypic and functional remodeling that critically influences NVU stability during both the acute phase of injury and the subsequent recovery period. Consequently, elucidating glia-mediated NVU remodeling represents a promising strategy for developing holistic neuroprotective and restorative approaches that extend beyond conventional neuron-targeted therapies.

This review synthesizes current knowledge on glial-driven NVU remodeling in stroke. An overview of major glial cell types and their roles in NVU homeostasis is first provided. Subsequently, glial responses specific to different phases and stroke types, the impact of key risk factors, and emerging glia-targeted therapeutic strategies are examined.

## 2 Glial roles in NVU homeostasis

### 2.1 Overview of glial cells

**Figure 1:**
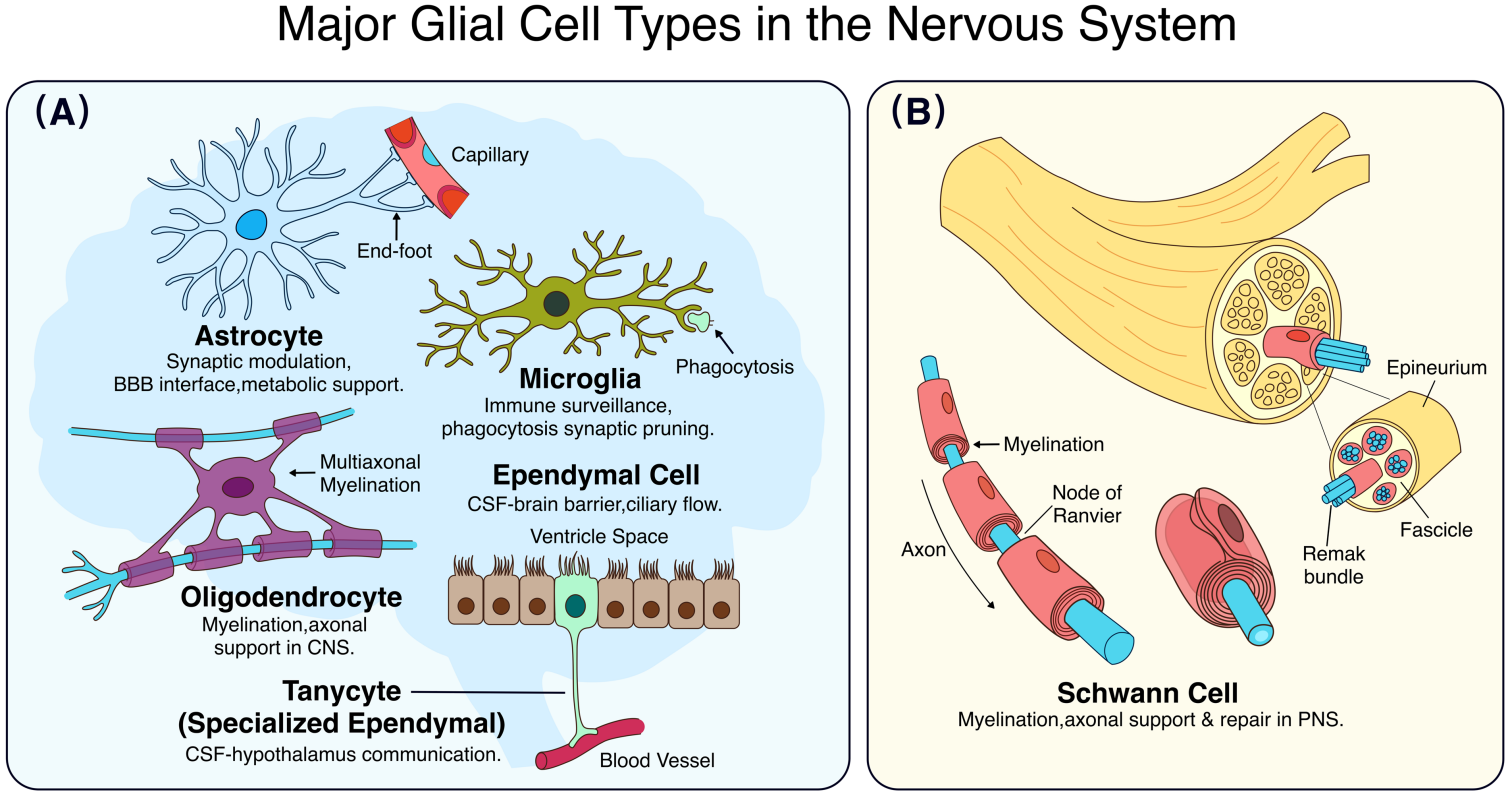
Schematic classification of major glial cells in the nervous system. This illustrative schematic summarizes the classification, characteristic morphology, and canonical functions of principal glial populations. **(A)** Central nervous system (CNS) glia. Astrocytes (blue) contact blood vessels via end-feet and regulate synaptic activity and blood–brain barrier (BBB) integrity. Microglia (green) mediate immune surveillance and phagocytosis (arrows). Oligodendrocytes (purple) myelinate multiple axons. Ependymal cells (brown) line the ventricles and regulate cerebrospinal fluid (CSF) flow, while tanycytes extend processes toward blood vessels to mediate CSF-hypothalamic communication. **(B)** Peripheral nervous system (PNS) glia. Schwann cells (red) form myelinating internodes separated by nodes of Ranvier or non-myelinating Remak bundles. A nerve cross-section shows fascicles surrounded by epineurium. BBB, blood–brain barrier; CSF, cerebrospinal fluid; CNS, central nervous system; PNS, peripheral nervous system.

Glial cells, owing to their plasticity and the ideal location at the interface between blood vessels and neurons, play a pivotal role in integrating and transmitting information within the neural network. Not only do they support brain function, but they also generate adaptive responses to nerve injury (Goetz and Sirko, 2023; Ferrarelli, 2017). Based on their morphology and functional roles, key central nervous system (CNS) glial cell types include astrocytes, microglia, oligodendrocytes, ependymal cells, and tanycytes, among others (Figure 1; Nampoothiri et al., 2022; Tsintzou et al., 2025).

Astrocytes are the predominant glial cells in the CNS and serve as crucial regulators of neural circuit function (Allen, 2013; Brandebura et al., 2023). They form the ‘tripartite synapse’ with neurons, participating in neurotransmitter recycling and modulating neuronal activity (Allen and Eroglu, 2017; Guttenplan et al., 2025; Weber and Barros, 2015). Astrocytes possess numerous fine processes that closely interact with both blood vessels and neurons, facilitating the delivery of glucose and oxygen from the bloodstream to active neurons (Attwell et al., 2010; Haydon and Carmignoto, 2006). They can be broadly classified into protoplasmic astrocytes in gray matter and fibrous astrocytes in white matter, exhibiting significant molecular heterogeneity across brain regions (Huang et al., 2020; Wang and Bordey, 2008). Region-specific transcription factors such as Nkx2.1, Lhx2, Foxg1, and Zic1 have been identified in shaping astrocyte molecular identity and function in different central nervous system domains.

Microglia represent the distinct population of resident immune cells in the CNS, originating from the hematopoietic lineage (Allen and Barres, 2009). They remain highly dynamic, continuously surveying the CNS even under physiological conditions (Davalos et al., 2005). Microglia are primarily engaged in phagocytic activity, regulating synaptic number and eliminating inappropriate connections (Brown and Neher, 2014; Vainchtein and Molofsky, 2020). They can also promote synaptogenesis and modulate neuronal activity (Li et al., 2012; Miyamoto et al., 2016; Weinhard et al., 2018). Their ability to sense neuronal activity enables precise regulation of phagocytosis, essential for maintaining brain health (Eyo and Molofsky, 2023).

Oligodendrocytes in the CNS produce myelin to form the myelin sheath, providing metabolic support to axons and regulating the speed of action potential conduction (Funfschilling et al., 2012). Oligodendrocyte precursor cells (OPCs) originate from the neural tube. Although myelination is highly plastic, it follows a predictable spatial and temporal pattern that continues into adulthood (Lebel et al., 2012; Richardson et al., 2006; Taylor and Monje, 2023). The development of oligodendrocytes and myelination is highly regulated by axonal surface ligands, secreted molecules, and axonal activity (Emery, 2010). Schwann cells (SCs) are the myelinating glia of the peripheral nervous system (PNS), arising from neural crest cells (Wanner et al., 2006). They provide essential structural and metabolic support to peripheral axons, a function that is evolutionarily conserved and operates independently of myelination (Nave and Werner, 2021; Taveggia and Feltri, 2022). SCs engage in metabolic coupling with the axonal compartment, supplying essential metabolites and cofactors (Nave, 2010). Following nerve injury, SCs undergo phenotypic reprogramming into repair-supportive cells, dedifferentiating, clearing debris, and forming Büngner bands to guide axonal regeneration (Jessen and Mirsky, 2016).

Ependymal cells line the ventricular surfaces with a ciliated epithelium. Mature ependymal cells exhibit limited regenerative capacity but possess the machinery for clearing substances from the cerebrospinal fluid (CSF), contributing to a metabolic barrier at the brain-CSF interface (Del, 2010; Groh et al., 2024). Tanycytes are a specialized subtype of ependymal cells in the hypothalamus, characterized by elongated processes that contact capillaries. They are involved in linking the CSF with the external basement membrane and play roles in neuroendocrine signaling and metabolic regulation (Rodriguez et al., 2010, 2019).

### 2.2 NVU structure and function

The concept of NVU represents a paradigm shift from an isolated to an integrated understanding of the interactions between neural cells and the vascular system in the brain, providing a critical theoretical framework for studying neurovascular physiology and pathology (Sun et al., 2021). The NVU is a functional multicellular assembly embedded within a shared microenvironment. It includes neurons, glial cells (e.g., astrocytes, oligodendrocytes, microglia), vascular cells (e.g., endothelial cells, pericytes, vascular smooth muscle cells, and extracellular matrix), and adjacent mural cells (Morrison and Filosa, 2019; Tam and Watts, 2010). The NVU has two core functions. First, it dynamically regulates cerebral blood flow (CBF) by coordinating vascular responses to neuronal activity, thus meeting the metabolic needs of active brain regions. Second, it plays a key role in BBB formation and maintenance and in modulating neuroimmune responses (Banks et al., 2015; Thurgur and Pinteaux, 2019). Together, these processes underlie neurovascular coupling-the precise, bidirectional coordination between neural activity and local hemodynamic changes—serving as a fundamental mechanism for maintaining cerebral homeostasis and supporting normal neurological function.

The intricate intercellular interactions within the NVU necessitate highly coordinated functions among its cellular components to maintain BBB homeostasis and enable effective neurovascular coupling (Kugler et al., 2021). Endothelial cells (EC) form a continuous monolayer lining the cerebral vasculature and are structurally and functionally specialized at the BBB. These cells exhibit reduced transcytosis and transcellular transport (O'Brown et al., 2019), upregulate the expression of tight junction proteins which restrict paracellular permeability (Hashimoto and Campbell, 2020; Sweeney et al., 2019), and effectively exclude the free diffusion of molecules larger than 400 kDa (Baghirov, 2025). Embedded within the shared basement membrane alongside ECs, mural cells—including pericytes and vascular smooth muscle cells (vSMCs)—contribute to vascular stability, provide structural support, and regulate vasomotor tone. Moreover, they play a critical role in BBB induction, maturation, and maintenance, thereby actively participating in NVU function. Mural cells, including pericytes and vascular smooth muscle cells, contribute to vascular stability, regulate vasomotor tone, and play critical roles in BBB induction, maturation, and maintenance (Henshall et al., 2015). Neurons within the NVU contribute to hemodynamic regulation by directly releasing vasoactive signaling molecules such as nitric oxide (NO) to modulate local cerebral blood flow. Additionally, neuronal activity influences vascular dynamics and BBB integrity through the release of neurotransmitters (Lacoste et al., 2014).

### 2.3 Glial support for neurons within the NVU

**Figure 2:**
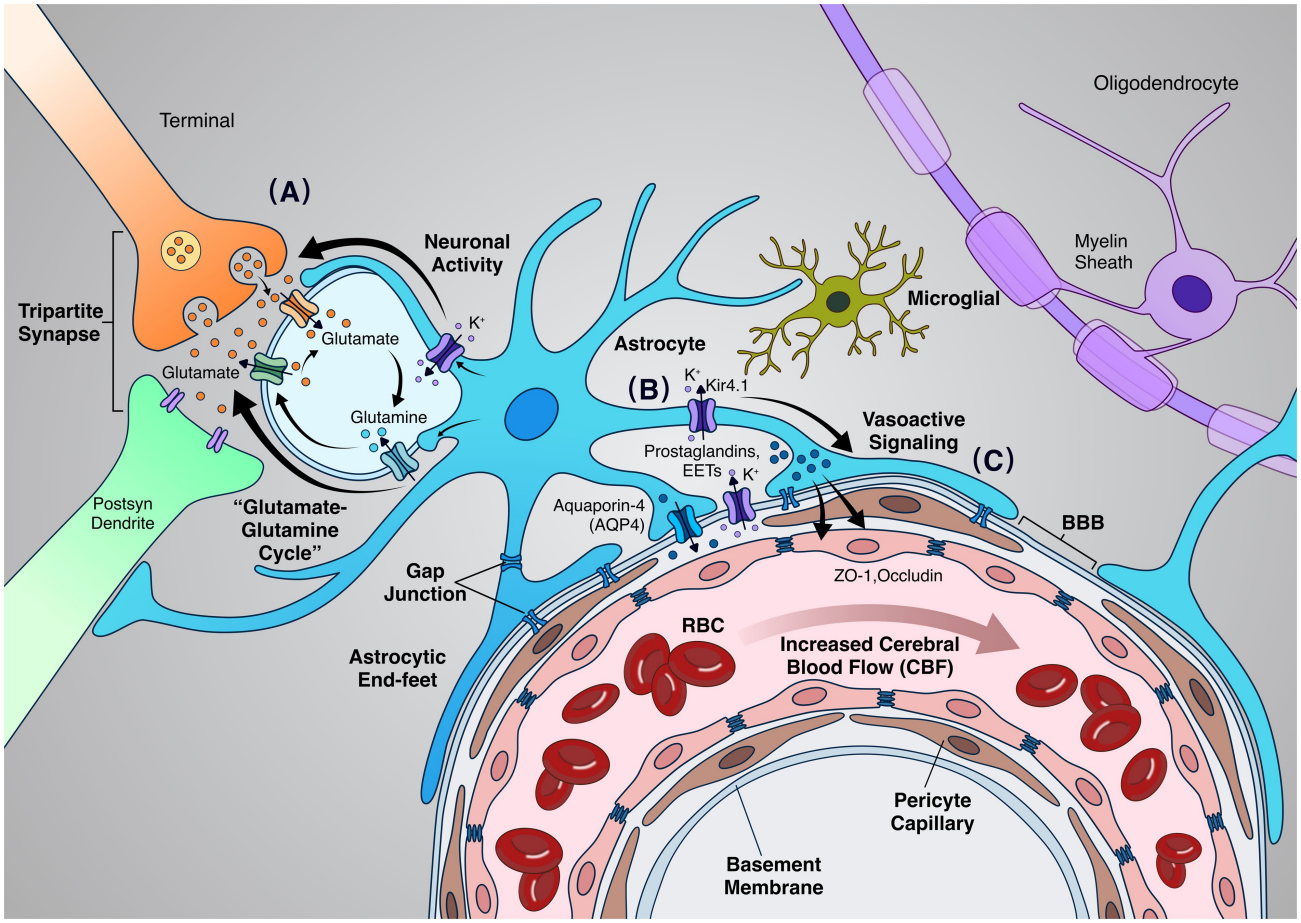
The key mechanisms of glia-mediated neurovascular coupling. **(A)** At the tripartite synapse, neuronal activity induces glutamate release, which is taken up by astrocytes and converted to glutamine, forming the glutamate-glutamine cycle (arrows indicate neurotransmitter flux). **(B)** Astrocytic signaling propagates to perivascular end-feet enriched in aquaporin-4 (AQP4) and K⁺ channels (e.g., Kir4.1), enabling ion and water homeostasis. Astrocytes release vasoactive mediators (e.g., prostaglandins, EETs, and K⁺) toward the vasculature (arrows indicate signal direction).**(C)** Vasoactive signals act on pericytes and endothelial cells at the blood–brain barrier (BBB), modulating vascular tone and increasing cerebral blood flow (CBF), as illustrated by red blood cell (RBC) movement within the capillary. AQP4, aquaporin-4; BBB, blood–brain barrier; CBF, cerebral blood flow; RBC, red blood cell; EETs, epoxyeicosatrienoic acids.

As mentioned above, the “tripartite synapse” structure formed by glial cells (Figure 2; Hillen et al., 2018; Ji et al., 2026), through which they exert dynamic regulatory functions in modulating neuronal activity. Alterations in their morphology and volume not only directly influence the homeostasis of the neural microenvironment but also trigger functional signal transmission effects (Deng et al., 2024). This process involves coordinated actions of multiple molecular mechanisms, including aquaporin-4 (AQP4)-mediated water transport (Gleiser et al., 2016), transient receptor potential vanilloid 4 (TRPV4) channel-regulated calcium influx (Jo et al., 2015), and ion transport systems such as connexin 43 (Cx43), potassium inwardly rectifying channel 4.1 (Kir4.1), and Na⁺/K⁺-ATPase in potassium homeostasis (Lafrenaye and Simard, 2019).

Beyond physical connections, glial cells functionally interact with the NVU by releasing gliotransmitters such as gamma-aminobutyric acid (GABA), glutamate, and cytokines to synchronize and modulate neural activity within synapses (Sibille et al., 2014; Zharikova et al., 2026). Additionally, glial cells terminate synaptic transmission and prevent excitotoxicity by clearing neurotransmitters from the synaptic cleft, and utilize the glutamate-glutamine cycle to shuttle neurotransmitters back to neurons, replenishing the functional neurotransmitter pool (Limon et al., 2021; Turner and Adamson, 2011).

### 2.4 Glial support for vasculature within the NVU

During vasculogenesis, glial precursor cells promote angiogenesis by expressing factors such as vascular endothelial growth factor (VEGF), Wnt, GPCR124, and TGF-β1 (Sato et al., 2022; Siqueira et al., 2018). Microglia play a critical role in vascular fusion by mediating the connection of endothelial tip cells via Notch1 and delta-like ligand 4 (dll4) expression (Outtz et al., 2011), and through tyrosine kinase with immunoglobulin-like and EGF-like domains 2 (Tie2) and neuropilin 1 (Nrp1) involvement in VEGF-mediated regulation of endothelial tip cell behavior (Fantin et al., 2010).

Following vascular formation, glial cells promote BBB maturation and stabilization through multiple mechanisms. Glial precursor cells secrete retinoic acid to enhance BBB stability and upregulate BBB-specific proteins including P-gp, occludin, and glutathione S-transferase-1(Mizee et al., 2013). Astrocytes contribute via Src-suppressed C kinase substrate (SSeCKS) mediated modulation of VEGF levels to promote tight junction formation (Wang Z. et al., 2020), and via angiotensin-converting enzyme 1 (ACE-1) catalyzed angiotensin II production to support BBB maturation and stabilize junctional proteins (Hatho Towo et al., 2026). Bidirectional crosstalk between endothelial and glial cells coordinates BBB development: glial precursor cells support endothelial cell maturation by inhibiting proliferation and reducing vascular permeability, while mature endothelial cells secrete VEGF-A to induce astrocyte differentiation and facilitate NVU assembly (Ashraf et al., 2020). Ependymal cells of the choroid plexus also form an integral component of the BBB system, with tight junctions between epithelial cells strictly regulating blood-CSF molecular exchange (Evans et al., 2020; Nelles and Hazrati, 2022).

In addition to angiogenesis, glial cells regulate CBF within the NVU by releasing vasoactive factors such as calcium ions, NO, arachidonic acid, and prostaglandins, which directly modulate local microvessel vasoconstriction and vasodilation (Biesecker et al., 2016; Magaki et al., 2018). Glial cells also indirectly promote vasodilation through interactions with pericytes (Nortley and Attwell, 2017). Calcium signaling serves as a pivotal mediator, enabling intercellular coordination within the glial cell network and facilitating cross-cellular communication with endothelial cells to jointly modulate vascular tone (Munoz et al., 2015).

In summary, glial cells are integral to NVU structure and function, actively regulating BBB integrity, neurovascular coupling, and synaptic transmission. Astrocytes form the tripartite synapse, recycle neurotransmitters, and couple neuronal activity to local blood flow via calcium-dependent signaling. Microglia support vascular fusion during development, maintain barrier function through phagocytic mechanisms, and continuously survey the CNS microenvironment. Oligodendrocytes provide metabolic support to axons via myelination, while ependymal cells and tanycytes contribute to CSF homeostasis and neuroendocrine regulation. However, several key issues remain to be elucidated. The mechanisms by which distinct glial subtypes coordinate their responses to maintain NVU homeostasis under physiological conditions warrant further investigation. The molecular signals governing the dynamic interactions between astrocytes, microglia, and vascular cells during NVU formation and maintenance remain poorly defined. Moreover, the extent to which region-specific glial heterogeneity influences NVU function across different brain areas requires systematic characterization. Addressing these knowledge gaps will be essential for understanding how glial dysfunction contributes to NVU pathology in stroke.

## 3 Roles of NVU-related glial cells after stroke

### 3.1 NVU dysfunction after intracerebral hemorrhage injury

Following intracerebral hemorrhage (ICH), the NVU suffers both primary and secondary injury (Sasaki et al., 2020). Primary injury results from the rapid increase in intracranial pressure and direct mechanical disruption of brain tissue (Bodmer et al., 2012). Concurrently, the hematoma releases hemoglobin and free iron, which catalyze the production of reactive oxygen species (ROS), leading to oxidative stress, lipid peroxidation, and inflammatory responses (Dang et al., 2017). These pathological processes directly injure NVU, and activate glial cells.

Oligodendrocytes, which are rich in iron, are particularly vulnerable to iron overload (Wan Y. et al., 2025), leading to their death and demyelination in white matter tracts (Kang and Yao, 2019). Astrocytes are enriched with neurotransmitter transporters, and impairment of their function disrupts the temporal dynamics of synaptic signaling, thereby compromising the normal operation of neural circuits (Scimemi, 2018). Furthermore, a study has shown that ICH enhances the proliferation of ependymal cells in the subventricular zone (SVZ) (Williamson et al., 2023). CXCL12 derived from the perivascular region surrounding the stroke lesion can signal to CXCR4-expressing cells in the SVZ, inducing the ectopic migration of newborn cells toward the site of injury.

**Figure 3:**
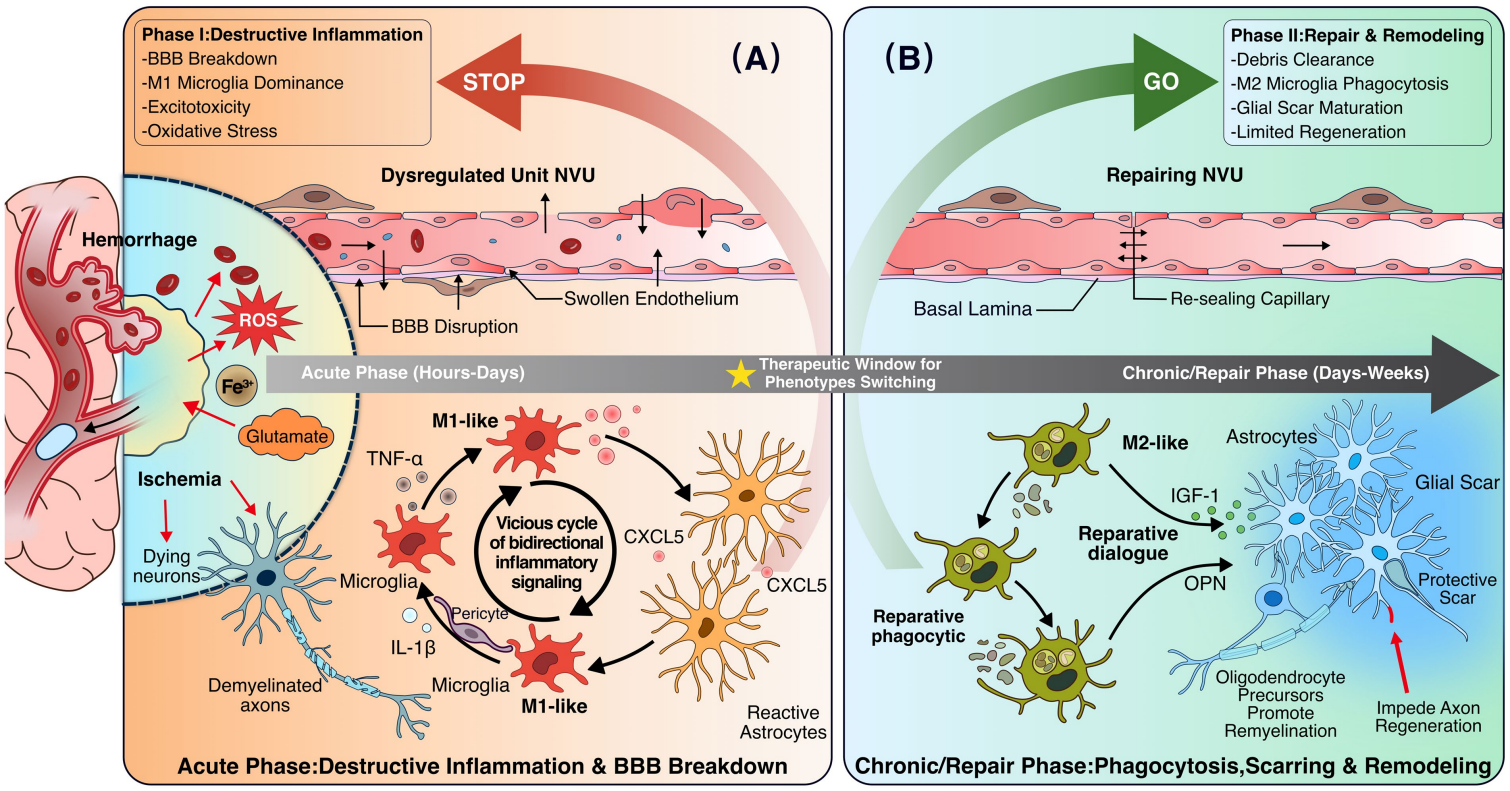
Pathological evolution of the post-stroke neurovascular unit mediated by glial cells. **(A)** Acute phase (hours-days). Ischemia and hemorrhage trigger blood–brain barrier (BBB) disruption, oxidative stress, and excitotoxicity. M1-like microglia release pro-inflammatory mediators (e.g., TNF-α, IL-1β), interacting with reactive astrocytes to form a feed-forward inflammatory loop (arrows), leading to NVU dysfunction. **(B)** Chronic/repair phase (days-weeks). Microglia shift toward an M2-like phenotype, promoting debris clearance and vascular repair via factors such as IGF-1 and OPN. Astrocytes contribute to glial scar formation, which limits inflammation but may impede axonal regeneration. Bidirectional signaling between microglia and astrocytes (cytokines and chemokines) underlies coordinated remodeling of the NVU. BBB, blood–brain barrier; NVU, neurovascular unit; TNF, tumor necrosis factor; IGF, insulin-like growth factor; OPN, osteopontin.

Microglia are the primary immune responders in ICH. In the early phase, microglia display a predominantly pro-inflammatory phenotype, characterized by the release of IL-1β, TNF-*α*, and ROS. In contrast, anti-inflammatory phenotypes associated with tissue repair are less prevalent during this acute stage. Consequently, therapeutic strategies often aim to suppress the pro-inflammatory activation of microglia while promoting their anti-inflammatory and tissue-repairing functions (Lan et al., 2017). Activated microglia release signaling molecules that modulate astrocyte function, while astrocytes reciprocally regulate microglial activation through the secretion of cytokines (e.g., IL-6, IL-1β, IL-10) and chemokines (e.g., CCL2, CXCL1, CXCL10, CXCL12), forming a bidirectional regulatory network (Figure 3). Although SCs primarily function in peripheral nerve repair, emerging evidence suggests that transplanted SCs contribute to remyelination and neuronal repair in hemorrhagic lesions, indicating potential roles in CNS repair (Wan et al., 2007).

During the chronic phase of ICH, glial scar formation evolves dynamically. Early microglial depletion disrupts astrocytic scar formation, exacerbates neutrophil infiltration, and impairs tissue repair. Conversely, microglial repopulation, driven by insulin-like growth factor 1 (IGF1) and osteopontin (OPN), activates mammalian target of rapamycin (mTOR) signaling to promote protective scar formation. In the chronic phase, however, astrocytic scars shift toward a detrimental phenotype, and delayed microglial modulation can partially reverse this effect, underscoring the critical importance of timing in microglia-astrocyte interaction-based therapies (Zheng et al., 2023).

### 3.2 NVU dysfunction after cerebral ischemic injury

Cerebral ischemia disrupts the supply of glucose and oxygen, inhibiting glycolysis and oxidative phosphorylation, leading to ATP depletion, intracellular acidosis, and ROS accumulation (Abramov et al., 2007). In the ischemic core, neurons undergo rapid and irreversible damage, resulting in infarction (Majumder, 2024). The surrounding penumbra, though hypoperfused, retains partial viability (Koukalova et al., 2024). However, it is susceptible to transient ischemic depolarizations (TIDs; Ren et al., 2025), which may progress to terminal depolarization and infarct expansion. Additionally, the progressive increase in extracellular glutamate within the first hour of ischemia exacerbates excitotoxic injury, and blockade of glutamate receptors has been shown to mitigate this process and reduce infarct volume.

In the acute phase of ischemic stroke, astrocytes exhibit greater resilience due to prolonged ATP maintenance (Lu and Wen, 2025), lower glutamate receptor density, and enhanced ion buffering and antioxidant capacity. Following permanent middle cerebral artery occlusion (MCAO), pericyte-derived trophic factors activate astrocytes, inducing upregulation of glial fibrillary acidic protein (GFAP; Li et al., 2008) and triggering reactive astrogliosis and glial scar formation in the peri-infarct region (Fernandez-Klett et al., 2013; Shibahara et al., 2020).

Microglia respond rapidly to ischemic insult. The Purinergic receptor P2Y, G-protein coupled, 12 (P2RY12), a marker associated with homeostatic microglia, plays a critical role in the rapid repair of the BBB following injury (Lou et al., 2016). Studies have shown that genetic ablation or pharmacological inhibition of P2RY12 significantly impairs the extension of microglial processes toward sites of vascular damage, thereby preventing effective BBB sealing. After stroke, microglia rapidly transition from a homeostatic to an activated state (Eldahshan et al., 2019; Rawlinson et al., 2020). Due to their high sensitivity to subtle BBB disruptions (Petersen et al., 2018), they are recruited to perivascular regions within 6 h of reperfusion and fully ensheath small vessels in the peri-infarct area by 24 h (Jolivel et al., 2015). Intracellular vesicles containing CD31-positive material have been observed in perivascular microglia, suggesting phagocytosis of brain endothelial cells, a phenomenon closely associated with blood–brain barrier disruption.

Astrocytes and microglia exhibit close interactions following stroke. Studies have shown that astrocyte-derived CXCL5 inhibits microglial phagocytosis of myelin debris, exacerbating white matter injury and cognitive impairment, whereas activated microglia in the acute phase can promote neuroinflammation and infarct expansion (Cao et al., 2023; Li T. et al., 2021).

During the chronic phase of ischemic stroke, the glial scar, while limiting inflammation and containing damage, can also inhibit axonal regeneration and impair functional recovery (Figure 3; Beck et al., 2008). The balance between beneficial and detrimental effects of glial scarring represents a critical therapeutic challenge.

Collectively, glial cells undergo profound phenotypic and functional remodeling across distinct temporal phases after both ischemic and hemorrhagic stroke, critically influencing NVU integrity and recovery outcomes. In ICH, oligodendrocytes are vulnerable to iron-induced ferroptosis, microglia exhibit early M1-dominant polarization, and reactive astrocytes form a bidirectional regulatory network. In ischemic stroke, astrocytes demonstrate greater resilience than neurons and form glial scars that limit inflammation but may impede repair, while microglia are rapidly recruited to perivascular regions and can exacerbate BBB disruption. The “two-phase spatiotemporal regulation” hypothesis proposed by our team provides a framework for time-dependent therapeutic targeting of microglial function, with the acute phase (24–72 h) focusing on suppressing pro-inflammatory activation and the recovery phase (after 3–7 days) enhancing phagocytic and pro-repair functions.

### 3.3 Biphasic modulation of microglial function after stroke

As introduced above, our “two-phase spatiotemporal regulation” hypothesis proposes precisely targeting the functional transition window of microglia: suppressing inflammation in the early phase to reduce infarct volume, and enhancing phagocytic capacity in the later phase to improve cognitive recovery. To further elaborate, the acute phase is defined as the first 24 to 72 h post-stroke, during which suppressing excessive pro-inflammatory microglial activation is the primary therapeutic goal. The chronic phase is defined as the period from 3 to 7 days extending into weeks post-stroke, during which the therapeutic strategy shifts toward enhancing the beneficial functions of microglia, particularly their phagocytic capacity for debris clearance and their polarization toward a pro-repair phenotype (Zheng et al., 2023).

The proposed biphasic intervention is mechanistically grounded in the temporal dynamics of microglial functional plasticity, and more importantly, in the recognition that each phase presents distinct therapeutic opportunities and vulnerabilities. During the acute phase, previous reviews have summarized that the core pathological driver is the rapid release of danger-associated molecular patterns (DAMPs) such as HMGB1, ATP, and S100 proteins from damaged cells (Chan et al., 2025; Shichita et al., 2014). These DAMPs activate pattern recognition receptors (TLR2, TLR4, P2X7) on microglia, triggering NF-κB and mitogen-activated protein kinase (MAPK) cascades that produce IL-1*β*, TNF-*α*, and ROS. Critically, this acute inflammatory response is a double-edged sword: while it contributes to secondary tissue damage, it also upregulates phagocytic receptors (CD36, TREM2) that are essential for subsequent debris clearance (Deczkowska et al., 2020; Woo et al., 2016). Therefore, the therapeutic goal in the acute phase is not to completely block microglial activation, which would abolish the beneficial priming of phagocytic machinery, but to attenuate the excessive pro-inflammatory output while preserving the early upregulation of clearance receptors. This nuanced modulation is what distinguishes our hypothesis from conventional anti-inflammatory approaches that non-selectively suppress microglial function.

During the recovery phase, the natural trajectory of microglial responses shifts toward repair, driven by the clearance of DAMPs, the emergence of resolving signals (annexin A1, resolvins, lipoxins), and the uptake of apoptotic cells (Sica and Mantovani, 2012). Studies have demonstrated that these signals activate STAT6 and peroxisome proliferator-activated receptor gamma (PPARγ), leading to a pro-repair microglial state that secretes IL-10, transforming growth factor beta (TGF-β), brain-derived neurotrophic factor (BDNF), and insulin-like growth factor 1 (IGF1), promoting tissue remodeling, neurogenesis, and angiogenesis (Cherry et al., 2014; Chhor et al., 2013). However, this endogenous repair program is often delayed or insufficient after severe stroke, particularly in the presence of comorbidities such as aging or diabetes. Consequently, the therapeutic goal in the recovery phase is to actively enhance this natural repair transition, for example, by administering IL-4, PPARγ agonists, or efferocytosis-promoting agents, rather than passively waiting for spontaneous recovery. Importantly, this enhancement must be timed after the acute inflammatory surge has subsided; premature intervention may inadvertently amplify inflammation, as the resolving signals require a permissive microenvironment that is only established after DAMP clearance (Van den Bossche et al., 2017). Thus, the two-phase framework is not merely a descriptive timeline of microglial phenotypes but a prescriptive strategy that aligns therapeutic actions with the evolving functional demands of the injured NVU.

This hypothesis fundamentally differs from the classical M1/M2 paradigm. Instead of static polarization states, the present hypothesis emphasizes temporal functional transitions and advocates for phase-specific modulation: suppressing acute inflammation without microglial depletion, followed by enhancing repair functions without over-activation (Hu et al., 2015). This approach recognizes that the same molecular pathway may play opposing roles across different phases. Preclinical evidence supports this timing-dependent strategy. In ischemic stroke, minocycline reduces infarct volume only when administered within 6 h, while IL-4 initiated after day 3 promotes long-term recovery (Liu et al., 2016; Lu et al., 2026). In ICH, early microglial depletion worsens outcomes, whereas repopulation at day 3–5 facilitates hematoma clearance (Zheng et al., 2023). These findings underscore that intervention timing is critical, dissociating the two-phase concept from a simplistic pro-inflammatory versus anti-inflammatory dichotomy.

However, it should be noted that most of this evidence comes from rodent models, and translational success has been limited. For example, while minocycline showed consistent efficacy in preclinical studies (Van den Bossche et al., 2017), a recent multicenter trial reported only modest benefits in select patient subgroups, highlighting the gap between animal models and human stroke. Furthermore, the optimal timing windows may vary depending on stroke severity, comorbidities, and individual genetic background, which have not been systematically investigated. Thus, while the two-phase framework is conceptually promising, its clinical applicability requires validation in more diverse and clinically relevant models.

Recent advances in single-cell and spatial transcriptomics have begun to unravel the remarkable heterogeneity of glial responses following stroke (Zucha et al., 2024). Single-cell RNA sequencing has identified disease-associated microglia and activated response microglia as distinct subsets beyond the classical M1/M2 paradigm in the ischemic brain (McDonough and Weinstein, 2025). Spatial transcriptomic analyses have further revealed region-specific astrocyte activation gradients, with peri-infarct regions exhibiting distinct molecular signatures compared to distal areas (Scott et al., 2024). These emerging technologies provide an unprecedented resolution for understanding glial heterogeneity and offer new opportunities for cell subtype-specific therapeutic targeting.

Potential risks warrant consideration. Premature phagocytic enhancement may exacerbate acute inflammation (Kigerl et al., 2009), while delayed anti-inflammatory intervention may impede debris clearance and scar formation. Complete microglial depletion at any stage is detrimental given their essential roles in BBB repair and synaptic maintenance. Thus, the goal is time-dependent modulation, attenuating but not ablating acute inflammation, and promoting but not overdriving repair. Several critical questions remain unresolved. The molecular switches governing the pro-inflammatory to pro-repair transition have yet to be identified. Strategies to preserve glial scar benefits while minimizing axonal regeneration hindrance require further development. Future studies should validate this hypothesis in clinically relevant models and translate these time-dependent strategies into patient stratification and personalized protocols.

In brief, stroke triggers distinct, phase-dependent glial remodeling: intracerebral hemorrhage features iron-driven oligodendrocyte injury and early M1 microglial polarization, while ischemic stroke involves astrocytic scar formation and perivascular microglial recruitment. Our two-phase spatiotemporal regulation hypothesis advocates precisely timed modulation-attenuating, but not ablating, pro-inflammatory activation in the acute phase to limit secondary damage, followed by actively enhancing, rather than passively awaiting, phagocytic clearance and pro-repair functions in the recovery phase to promote tissue restoration.

## 4 Glia mediated NVU dysfunction as a convergence point for stroke risk factors

Current evidence has clearly established that multiple risk factors, such as hypertension, hyperglycemia and aging, significantly increase the risk of both ischemic and hemorrhagic stroke. Notably, these risk factors do not act in isolation on blood vessels or neurons (Cai et al., 2017; Kozniewska and Aleksandrowicz, 2026; Raut and Cucullo, 2025); rather, they broadly remodel the NVU. Given the central role of glial cells in maintaining NVU homeostasis, regulating blood–brain barrier integrity, and mediating neuroinflammation, we propose that stroke risk factors may predispose the NVU to a “pre-vulnerable state” by impairing glial cell function in advance. Consequently, when a cerebrovascular insult occurs, the NVU’s compensatory capacity is drastically compromised, leading to markedly exacerbated injury.

### 4.1 Hypertension

Hypertension induces progressive glial dysfunction that precedes overt cerebrovascular events, primarily through chronic hemodynamic stress and sustained cerebral hypoperfusion (Lewington et al., 2002). The most established glial alterations include astrocytic AQP4 mislocalization and microglial sensitization, both of which have been consistently documented in animal models and human hypertensive brains (Verma and Feng Earley, 2025; Yamagata, 2012).

Chronic hypertension disrupts the polarized perivascular localization of astrocytic AQP4, impairing glymphatic fluid exchange and compromising BBB mechanical integrity. Concurrently, microglia adopt a sensitized phenotype driven by angiotensin II signaling, sustaining a pro-inflammatory milieu through persistent release of IL-1β, TNF-*α*, and ROS (Cheng and Correia, 2022). These mechanisms are well supported by multiple independent studies.

Whether hypertension directly causes astrocytic endfeet retraction or primarily acts through vascular remodeling remains less clear. Additionally, the extent to which impaired neurovascular coupling precedes or follows glial dysfunction in humans requires further validation (Li et al., 2022).

### 4.2 Hyperglycemia

Chronic hyperglycemia disrupts glial homeostasis through three convergent pathways: chronic oxidative stress, metabolic reprogramming, and impaired white matter support.

In astrocytes, sustained high glucose drives AGE/RAGE signaling, activating downstream NF-κB and NADPH oxidase pathways, which amplifies oxidative stress and impairs glutamate uptake and potassium buffering (Rivera-Aponte et al., 2020). This cascade has been consistently replicated across *in vitro* and *in vivo* models. In microglia, chronic hyperglycemia induces a shift toward aerobic glycolysis (even under normoxic conditions), favoring a pro-inflammatory phenotype characterized by elevated IL-1β, TNF-α, and iNOS (Cheng et al., 2021; Hamanaka et al., 2018). Furthermore, hyperglycemia suppresses OPC differentiation via enhanced NADPH oxidase (NOX) activity and diminished neurotrophic support, leading to white matter compromise (Shen et al., 2017; Wang Y. et al., 2025).

The causal role of microglial metabolic reprogramming in NVU dysfunction remains debated, as it is unclear whether this shift is a primary pathological driver or an adaptive response to the hyperglycemic milieu. Moreover, the precise molecular switch governing the transition from physiological to pathological glycolysis in microglia has yet to be conclusively identified.

### 4.3 Aging

The detrimental effects of aging on NVU function are mediated by specific, well-documented glial cell changes, rather than by generalized or speculative age-related alterations.

Aged microglia adopt a senescence-associated secretory phenotype (SASP), characterized by reduced phagocytic activity, impaired resolution of inflammation, and sustained secretion of IL-6, TNF-α, and matrix metalloproteinases (Baixauli-Martín et al., 2025; Gross et al., 2025). This phenotype has been validated across multiple species and is directly linked to worsened stroke outcomes. Aged astrocytes exhibit functional senescence marked by decreased expression and membrane localization of excitatory amino acid transporter 2 (EAAT2), impairing glutamate clearance and lowering the threshold for excitotoxicity (Russo et al., 2025; Sharma et al., 2019). Additionally, ependymal cells and tanycytes show age-related structural disorganization and diminished ciliary motility, compromising CSF flow and perivascular clearance (Weissleder et al., 2019). Each of these glial alterations has been directly demonstrated to impair NVU function, by disrupting either metabolic support, fluid exchange, or neuroimmune regulation.

The possibility of pharmacologically reversing SASP in microglia remains unproven, and it is unclear whether such an intervention would restore NVU function without unintended adverse effects. Moreover, direct evidence establishing a causal link between ependymal dysfunction and NVU impairment in the aging human brain is still lacking.

Thus, hypertension, hyperglycemia, and aging converge on glial dysfunction, creating a “pre-vulnerable state” that markedly exacerbates stroke injury. Hypertension induces astrocytic AQP4 mislocalization and pro-inflammatory microglial activation. Hyperglycemia drives AGE/RAGE signaling, microglial metabolic reprogramming, and OPC suppression. Aging leads to microglial SASP, astrocytic EAAT2 decline, and ependymal/tanycyte dysfunction. These risk factors do not act in isolation but synergistically compromise NVU integrity. Several important issues remain to be addressed. The potential for early intervention targeting glial dysfunction in at-risk populations to prevent or mitigate NVU vulnerability before stroke onset remains to be determined. Moreover, the mechanisms by which multiple comorbid risk factors synergistically impact glial function and NVU integrity warrant systematic investigation. Elucidating these mechanisms will be crucial for developing preventive strategies for vulnerable populations.

## 5 Targeting glia mediated neurovascular unit dysfunction to intervene in stroke

Glia mediated dysfunction of the NVU represents a central pathological hallmark of both ischemic and hemorrhagic stroke. Precise targeting of glia-driven NVU dysregulation holds significant promise as a therapeutic strategy for stroke intervention.

In hemorrhagic stroke, the lysis of red blood cells releases excessive free iron, which triggers ferroptosis and leads to lipid peroxidation and functional impairment in astrocytes and microglia, thereby exacerbating NVU disruption. Iron chelators such as deferoxamine (DFO) and ferroptosis inhibitors including Ferrostatin-1 and Liproxstatin-1 not only alleviate iron-induced toxicity but also significantly attenuate glia-mediated NVU damage (Fei et al., 2024; Huang et al., 2022; Sun et al., 2016). Furthermore, pharmacological upregulation of glutathione peroxidase 4 (GPX4) in astrocytes enhances their antioxidant capacity, thereby preserving NVU integrity following intracerebral hemorrhage (Yang Q. et al., 2025).

In ischemic stroke, activated astrocytes and microglia compromise BBB integrity, exacerbate neuroinflammation, and impair cerebral blood flow regulation. Preclinical studies have demonstrated that pharmacological agents such as minocycline and fingolimod can attenuate brain injury by modulating the activation states of glial cells, thereby effectively preserving NVU integrity and function (Du et al., 2019; Shang et al., 2020).

Recent studies have further elucidated the critical roles of long non-coding RNA (lncRNA) and epigenetic regulators in the fine-tuned modulation of glial responses within the NVU. For instance, miR-155 promotes microglial polarization toward the proinflammatory M1 phenotype (Chen et al., 2023), whereas lncRNA MALAT1 contribute to astrocyte-mediated vascular protection (Ouyang et al., 2024). Therapeutic intervention targeting these molecular switches via antisense oligonucleotides, exosome-based delivery systems, or small-molecule modulators may represent a next-generation approach for precision therapy in stroke.

Among the numerous glia-targeting compounds listed in Tables 1, 2, several therapeutic classes stand out as particularly promising due to their well-defined molecular targets, consistent preclinical efficacy, and potential for clinical translation. These include PPAR agonists, Nrf2 activators, and TLR4 inhibitors.

**Table 1:** Therapeutic modulation of glial cells to improve relevant signaling pathways in the NVU after hemorrhagic stroke.

Targeting drug/compound	Targeting signaling pathway/receptor	Major targeting glia cells	Major targeting NVU	Authors	Citations
PPAR agonist					
Fenofibrate	PPARα	Astrocyte	Neurons	Wang et al.	(Wang et al., 2017)
Rosiglitazone	JNK/STAT3	Microglia	Neurons	Fei et al.	(Chao et al., 2023)
Nrf2 activator					
TRIOL	Nrf2	Microglia	Neurons	Wu et al.	(Wei et al., 2025)
Omaveloxolone	Nrf2	Microglia	Neurons	Hu et al.	(Hu et al., 2022)
TLR4/NF-κB inhibitor					
AQP2	TLR4/NFκB-p65	Astrocyte	Neurons	Deng et al.	(Deng et al., 2022)
Dexmedetomidine	NF-κB	Microglia	Vasculature	Guo et al.	(Guo et al., 2022)
Naoxueshu oral liquid	TLR4/MyD88/NF-κB	Microglia	Vasculature	Li et al.	(Li R. et al., 2024)
HO-1	NF-κB	Microglia	Neurons	Chen et al.	(Chen W. et al., 2025)
Xanthotoxol	NF-κB	Microglia	Neurons	Zhu et al.	(Zhu et al., 2023)
Ferroptosis / iron metabolism					
Lipocalin-2	FTL	Microglia	Neurons	Fei et al.	(Fei et al., 2024)
Atorvastatin	Lipocalin-2	Microglia	Neurons	Wang et al.	(Wang G. et al., 2025)
Other / mixed mechanisms					
Fractalkine	CD163/HO-1	Microglia	Vasculature	You et al.	(You et al., 2022)
LiCl	GSK-3β	Microglia	Neurons and Vasculature	Li et al.	(Li et al., 2019)
Resveratrol	Sirt3	Microglia	Neurons	Sun et al.	(Sun et al., 2023)
APN	Drp1	Astrocyte	Neurons	Wu et al.	(Wu et al., 2023)
Protocatechuic acid	mTOR	Microglia	Neurons	Xi et al.	(Xi et al., 2021)
Sesamin	p44/42 MAPK	Microglia	Neurons	Ohnishi et al.	(Ohnishi et al., 2013)
Curcumin	p38MAPK/PKC	Microglia	Neurons	Yang et al.	(Yang et al., 2014)
Didymin	Asc/Caspase-1/GSDMD	Microglia	Neurons	Gu et al.	(Gu et al., 2022)

**Table 2:** Therapeutic modulation of glial cells to improve relevant signaling pathways in the NVU after ischemic stroke.

Targeting drug/compound	Targeting signaling pathway/receptor	Major targeting glia cells	Major targeting NVU	Authors	Citations
PPAR agonist					
Oleoylethanolamide	PPARα	Microglia	Neurons	Li et al.	(Li et al., 2023b)
Agomelatine	PPARγ	Oligodendrocyte	Neurons	Wang et al.	(Wang et al., 2024)
Artemisinin	PPARγ	Microglia	Neurons	Xu et al.	(Li et al., 2025)
Ginkgetin	PPARγ	Microglia	Neurons	Tang et al.	(Tang et al., 2022)
Astragaloside IV	PPARγ	Microglia	Neurons and Vasculature	Li et al.	(Li L. et al., 2021)
Oleoylethanolamide	PPARα	Oligodendrocyte	Neurons	Zhou et al.	(Zhuo et al., 2023)
Nrf2 activator					
Tert-butylhydroquinone	Nrf2	Astrocyte	Vasculature	Chen et al.	(Chen Y. et al., 2019)
TLR4/NF-κB inhibitor					
Cottonseed oil	TLR4/NF-κB	Astrocyte and Microglia	Neurons and Vasculature	Liu et al.	(Liu et al., 2020)
Poliumoside	JAK/STAT3	Microglia	Vasculature	Gao et al.	(Gao et al., 2025b)
Qingda granule	TLR4/NF-κB/NLRP3	Microglia	Neurons	Cai et al.	(Cai et al., 2024)
FGF21	NF-κB and PPAR-γ	Microglia	Neurons	Wang et al.	(Wang D. et al., 2020)
Buyang Huanwu Decoction	NFκB/CREB	Astrocyte and Microglia	Neurons and Vasculature	Li et al.	(Li et al., 2024b)
NKILA	NF-κB	Astrocyte	Neurons	Gao et al.	(Gao et al., 2021)
Analgecine	TLR4/MyD88	Microglia	Neurons	Yang et al.	(Yang et al., 2021)
Acteoside	HMGB1/TLR4/NLRP3	Microglia	Vasculature	Liao et al.	(Liao et al., 2024)
Quercetin	PI3K/Akt/NF-κB	Microglia	Neurons and Vasculature	Li et al.	(Li L. et al., 2023)
Shikonin	NOD2/RIP2/NF-κB	Microglia	Neurons	Wang et al.	(Yang et al., 2024)
Gypenoside	STAT-3/HIF1-α and TLR-4/NF-κB/HIF1-α	Microglia	Neurons	Xia et al.	(Xia et al., 2024)
Mbnl1	NF-κB	Microglia	Neurons	Xu et al.	(Xu et al., 2025)
HSYA	HMGB1/NF-κB	Microglia	Neurons	Yao et al.	(Yao et al., 2025)
Meisoindigo	TLR4/NF-κB	Microglia	Neurons	Ye et al.	(Ye et al., 2019)
Sphk1	TRAF2/NF-κB	Microglia	Neurons	Su et al.	(Su et al., 2017)
Mogroside V	TLR4/TRADD	Astrocyte	Neurons	Chen et al.	(Chen M. et al., 2025)
Immunomodulator / microglial polarization					
Minocycline	STAT1/STAT6	Microglia	Neurons	Lu et al.	(Lu et al., 2021)
PI3K/Akt pathway activators					
Scutellarin	PI3K-Akt	Astrocyte and Microglia	Neurons	Chen et al.	(Chen H. et al., 2025)
Honokiol	Akt	Oligodendrocyte	Neurons	Zhang et al.	(Zhang et al., 2023)
Esketamine	AKT	Microglia	Neurons	Wang et al.	(Gao et al., 2025a)
Epigenetic / RNA regulators					
miR-124	DLL4	Astrocyte	Neurons	Guo et al.	(Guo et al., 2023)
METTL14	KAT3B-STING	Microglia	Neurons	Li et al.	(Li et al., 2023a)
SNHG8	miR-449c-5p/SIRT1/FoxO1	Microglia	Vasculature	Zhang et al.	(Zhang et al., 2022)
Curcumin	microRNA-205-5p/KLF2/ATF2	Microglia	Neurons	Cao et al.	(Cao and Pu, 2025)
Resveratrol	miR-155	Microglia	Neurons	Ma et al.	(Ma et al., 2020)
PRMT8	Lin28a	Microglia	Neurons	Zheng et al.	(Zheng et al., 2022)
Other / mixed mechanisms					
Icaritin	GPER	Astrocyte	Neurons	Su et al.	(Su et al., 2025)
NAT10 inhibitors	Timp1 mRNA	Astrocyte	Neurons	Li et al.	(Yang L. et al., 2025)
Echinacoside	HIF-1α/LDHA	Astrocyte	Neurons and Vasculature	Liao et al.	(Liao et al., 2025)
Adjudin	Sirt3-Foxo3a	Astrocyte	Neurons and Vasculature	Yang et al.	(Yang et al., 2017)
BM-MSCs	AKT/mTOR	Astrocyte	Vasculature	Zhang et al.	(Zhang et al., 2017)
3,3,5 triiodo-l-thyronine	FAO	Astrocyte	Neurons	Sayre et al.	(Sayre et al., 2017)
Botch	Notch	Astrocyte and Microglia	Neurons	Gong et al.	(Gong et al., 2024)
Galangin	RhoA/ROCK/LIMK	Astrocyte	Neurons	Liu et al.	(Liu et al., 2025)
cTBS	TSP1	Astrocyte	Neurons	Hu et al.	(Hu et al., 2025)
Propofol	AQP4	Astrocyte	Neurons	Zhu et al.	(Zhu et al., 2009)
Avicularin	NLRP3	Microglia	Neurons	Shi et al.	(Shi et al., 2024)
ETV5	HACE1	Microglia	Neurons	Meng et al.	(Meng et al., 2025)
Ginsenoside Rb1	RET-ROS	Astrocyte	Neurons	Ni et al.	(Ni et al., 2022)
MLIF	eEF1A1	Microglia	Neurons	Zhang et al.	(Zhang D. et al., 2019)
RBM3	zr17-2	Microglia	Neurons	Zhao et al.	(Zhao et al., 2024)
Jasminoidin	PASK-EEF1A1	Microglia	Vasculature	Wu et al.	(Wu et al., 2024)
Astragaloside IV	AMPK	Microglia	Vasculature	Li et al.	(Li et al., 2024a)
Minocycline	EMB/MCT4/STING	Microglia	Neurons	Cheng et al.	(Cheng et al., 2025)
Celastrol	IL-33/ST2	Microglia	Neurons	Jiang et al.	(Jiang et al., 2018)
Ginsenoside Rg1	Pink1/Parkin	Microglia	Neurons	Sui et al.	(Sui et al., 2024)
COP1	C/EBPβ	Microglia	Neurons	He et al.	(He et al., 2022)
PD-1	MAPK	Microglia	Neurons	Hu et al.	(Huang et al., 2024)

PPAR agonists (e.g., fenofibrate, rosiglitazone, oleoylethanolamide) exert broad anti-inflammatory and metabolic effects (Montaigne et al., 2021). In hemorrhagic stroke, fenofibrate promotes astrocyte proliferation expression, while rosiglitazone suppresses M1 microglial polarization via JNK/STAT3 (Chao et al., 2023). In ischemic stroke, oleoylethanolamide enhances microglial M2 polarization and oligodendrocyte differentiation (Li et al., 2023b). These agents are typically administered intraperitoneally or orally in preclinical models, showing good brain penetration. However, clinical translation faces challenges including potential systemic metabolic side effects (e.g., fluid retention with rosiglitazone) and the need for stroke-specific dosing regimens distinct from those used for diabetes.

Nrf2 activators (e.g., omaveloxolone, tert-butylhydroquinone, TRIOL) enhance antioxidant capacity and promote microglial phagocytosis of hematoma debris (Wei et al., 2025). Omaveloxolone given intraperitoneally post-ICH shifts microglia toward a reparative phenotype and reduces secondary brain injury (Hu et al., 2022). Tert-butylhydroquinone administered orally enhances angiogenesis and astrocyte activation after ischemia (Chen Y. et al., 2019). Key translational hurdles include the narrow therapeutic window for Nrf2 activation (excessive or sustained activation may interfere with redox signaling needed for repair), as well as limited data on chronic safety in stroke populations.

TLR4/NF-κB pathway inhibitors (e.g., AQP2-targeting agents, poliumoside, Qingda granule) directly suppress pro-inflammatory glial activation (Bai et al., 2025). Poliumoside, given intraperitoneally after ischemic stroke, reduces BBB disruption and promotes microglial M2 polarization (Wang et al., 2025). Qingda granule (oral formulation) inhibits TLR4/NF-κB/NLRP3 signaling in microglia (Cai et al., 2024). Despite robust preclinical efficacy, challenges include achieving sufficient brain exposure with oral delivery and avoiding broad immunosuppression that could impair debris clearance or increase infection risk.

Other notable classes include ferroptosis inhibitors (e.g., lipocalin-2 blockade, Ferrostatin-1) for hemorrhagic stroke, which show strong efficacy but require further development of brain-permeable, safe small molecules, and RNA-based modulators (e.g., miR-124, NKILA) that offer high specificity but face delivery and stability hurdles (Du et al., 2025; Yan et al., 2025).

Beyond traditional pharmacological interventions, emerging concepts of the NVU as a dynamic multi-cellular and immune-interactive system have opened new therapeutic avenues. Extracellular vesicles (EVs), including exosomes derived from glial cells, mediate intercellular communication within the NVU by transferring proteins, lipids, and RNAs between cell types (Negah et al., 2025). Astrocyte-derived EVs have been reported to protect neurons against ischemic injury, while microglial EVs can propagate inflammatory signals (Wan H. et al., 2025). Conversely, mesenchymal stem cell-derived EVs are being explored as cell-free therapeutic agents for stroke (Arbabian et al., 2026). Additionally, recent advances in neuroimmune interactions have revealed that glial cells orchestrate peripheral immune cell infiltration and function after stroke, suggesting that modulating peripheral immunity could influence NVU repair (Kanazawa and Hatakeyama, 2025). Advanced *in vitro* models, such as NVU-on-chip and brain organoids, are now being developed to recapitulate the multicellular NVU microenvironment and enable high-throughput screening of glia-targeted compounds, accelerating the translation of these therapies (Chim et al., 2024).

In summary, PPAR agonists, Nrf2 activators, and TLR4 inhibitors represent the most advanced glia-targeting strategies for NVU protection in stroke. Their preclinical efficacy is well documented across multiple models, but translation will require optimization of delivery routes, timing, and patient selection to balance benefit against potential off-target effects. Combination with recanalization therapies and validation in comorbid populations (aged, hypertensive, diabetic) are critical next steps.

## 6 Conclusion and perspective

Since the introduction of the NVU concept, it has become clear that glial cells function beyond classical support, serving as central regulators of NVU homeostasis and pathology in stroke. Astrocytes, microglia, oligodendrocytes, and specialized ependymal derivatives respond dynamically to ischemic or hemorrhagic insults through coordinated alterations in morphology, metabolism, and intercellular signaling. These changes collectively modulate blood brain barrier integrity, neuroinflammatory responses, oxidative stress, and neural repair processes.

A key theme emerging from this review is that glial responses are not monolithic but instead exhibit remarkable spatiotemporal heterogeneity. In the acute phase of stroke, reactive astrocytes form glial scars that initially contain damage but may later impede repair. Concurrently, microglia rapidly transition from a homeostatic to a proinflammatory state, contributing to secondary injury while also priming the phagocytic machinery essential for debris clearance. In the recovery phase, glial functions shift toward tissue remodeling, neurogenesis, and angiogenesis. Our proposed “two phase spatiotemporal regulation” hypothesis captures this duality and provides a framework for time dependent therapeutic targeting. This approach involves attenuating, but not abolishing, proinflammatory activation in the acute phase, followed by actively enhancing phagocytic clearance and pro repair functions in the recovery phase.

Preclinical studies indicate that targeted modulation of glial activation using immunomodulatory agents or RNA based interventions can effectively preserve NVU structure and function, leading to improved functional outcomes in animal models of stroke. These findings collectively suggest that glia centered strategies hold promise for extending the therapeutic window beyond conventional neuron focused approaches.

Looking forward, understanding glial mediated NVU remodeling can inform therapy development in several specific ways. First, the recognition of phase dependent glial functional transitions calls for the development of adaptive therapeutic regimens, in which drug type, dose, and duration are tailored to the evolving NVU microenvironment rather than fixed protocols. Second, the identification of region specific transcription factors, along with evidence of glial heterogeneity across brain areas, highlights the need for region targeted interventions. A therapy effective in cortical glia may not translate to white matter or subcortical structures. Third, the convergence of common stroke risk factors, including hypertension, hyperglycemia, and aging, on glial dysfunction suggests that preventive glia targeted strategies in at risk populations could mitigate NVU vulnerability before stroke onset. Fourth, the successful translation of glia modulating agents will require optimization of delivery routes, such as intranasal or exosome based approaches, to enhance brain penetration. It will also require refinement of dosing windows to align with biphasic responses and validation in comorbid animal models that recapitulate the aged, hypertensive, or diabetic human condition.

Critical challenges remain. The molecular switches governing the proinflammatory to pro repair transition in microglia have yet to be fully elucidated. The dual role of glial scars, beneficial containment versus detrimental regeneration barrier, demands strategies that preserve protective functions while mitigating inhibitory effects on axonal repair. Furthermore, the spatiotemporal heterogeneity of glial responses across distinct brain regions and disease phases requires systematic characterization, ideally through single cell and spatial transcriptomic approaches integrated with high resolution imaging. Moreover, many studies rely on pharmacological inhibitors with off-target effects or genetic models with developmental compensation, which may confound interpretation. The predominant use of young, healthy male animals in preclinical stroke research also limits generalizability to the clinical population, which is often aged and comorbid. Future studies should prioritize rigorous experimental designs, including validation of findings across multiple models, systematic reporting of negative results, and increased use of both sexes and aged animals to enhance translational relevance.

In conclusion, glial cells have emerged as central hubs in NVU remodeling after stroke. By embracing the complexity and dynamics of glial responses and translating these insights into phase dependent, region aware, and risk stratified therapeutic strategies, the field can move beyond neuron centric paradigms toward more holistic, mechanism driven interventions for NVU protection and functional recovery in stroke patients.
